# Distribution and Genetic Diversity of *Salmonella enterica* in the Upper Suwannee River

**DOI:** 10.1155/2011/461321

**Published:** 2011-12-13

**Authors:** Masoumeh Rajabi, Melissa Jones, Michael Hubbard, Gary Rodrick, Anita C. Wright

**Affiliations:** Food Science and Human Nutrition Department, University of Florida, Gainesville, FL 32611, USA

## Abstract

The Suwannee River spans the Florida/Georgia border to the Gulf of Mexico, and contributes to regional irrigation and recreational activities. Association of *Salmonella enterica* with these resources may result in the contamination of produce and disease outbreaks. Therefore, surface water was examined for the distribution of *S. enterica* at multiple time points from 4 sites on the upper Suwannee River. Isolates were confirmed by detection of the *invA* gene, and 96% of all samples were positive for the bacterium. Most probable number enumeration ranged from <18 to 5400 MPN/100 mL. Genetic diversity of these isolates (n=110) was compared to other environmental (n=47) or clinical (n=28) strains and to an online library (n=314) using DiversiLab rep-PCR. All strains showed >60% similarity and distributed into 16 rep-PCR genogroups. Most (74%) of the Suwannee River isolates were clustered into two genogroups that were comprised almost exclusively (97%) of just these isolates. Conversely, 85% of the clinical reference strains clustered into other genogroups. However, some Suwannee River isolates (12%) were clustered with these primarily clinically-associated genogroups, supporting the hypothesis that river water can serve as a disease reservoir and that pathogenic strains may persist or possibly originate from environmental sources.

## 1. Introduction

Nontyphoidal salmonellosis is the leading cause of bacterial foodborne illness in the US and contributed to approximately 33% of all foodborne-related deaths in 2009 [1]. The disease is characterized by a gastroenteritis that is associated with a wide range (>2500) of *Salmonella enterica* serotypes [2]. Historically, disease reservoirs for *Salmonella *were primarily attributed to contaminated poultry meat and eggs, but other sources include soil, factory surfaces, animal feces, and raw meats [3, 4]. More recently, orange juice [5–8] and other produce products [9–12] have been increasingly implicated as the source of salmonellosis outbreaks. Moreover, the number of cases per outbreak is greater for vegetables than for any other product [13].

Irrigation water may play an important role in contaminating soil and produce with *Salmonella* [14, 15]. Storm water runoff and septic tanks are known contributors of pathogens to surface water [16, 17], but rain events may also aid in the transport of pathogens from environmental sources in forested and grassed buffer zones into farm ponds [18]. Laboratory assays have demonstrated the potentiality of pathogen uptake through the roots [19] and flowers of edible plants [20], and *Salmonella* from irrigation sources has been shown to adhere to plants and survive for long periods of time [21].

The Suwannee River is a central feature of the Suwannee River watershed and is the largest source of free-flowing fresh water to the Gulf of Mexico [22]. The region is characterized by relatively minimal human impact and spans the coastal plains of southern Georgia and north central Florida. A recent survey of surface water collected within the southwest Georgia portion of the watershed reported the presence of *S. enterica *[17]. This region is considered a “hot spot” for environmental sources of salmonellosis, as case rates within this region were 1.5 times higher than the national average [14, 23], and river water collected locally over a 12-month period was positive for *S. enterica *in 79.2% of samples [17]. *S. enterica *densities directly correlated with water temperature (*r* = 0.49; *P* < 0.05) and precipitation levels (*r* = 0.68; *P* < 0.05) and also increased 62% in summer compared to spring months. This survey also identified serovars with environmental sources that were associated with disease in this region. However, the genetic relationship of strains from clinical and environmental sources was not examined.

*S. enterica *isolates from aquatic environments show a wider diversity of serotypes than those recovered from animal sources [24–26], but the genetic diversity and distribution of *S. enterica* from environmental sources is still relatively unknown. Therefore, the present study examined the genetic profiles of *S. enterica *recovered from the Suwannee River to those of strains from clinical and other environmental sources. The DiversiLab repetitive extragenic palindromic PCR (rep-PCR) analysis was used to evaluate genetic similarity of *Salmonella *strains because it shows enhanced discrimination compared to other methods such as pulsed field gel electrophoresis [27–29]. Results showed that *S. enterica* from Suwannee River surface water samples comprised a diverse population that was genetically distinct from strains from other environmental sources and from most strains of clinical origin. This research establishes an *S. enterica *database that should serve as reference for routine monitoring and source tracking in future outbreaks. 

## 2. Materials and Methods

### 2.1. *S. enterica* Strains and Culture Conditions

*S. enterica *isolates were recovered from surface water samples of the Suwannee River (*n* = 110), as described below. Reference strains included a total of 186 *S. enterica* isolates from both clinical (*n* = 31) and environmental (*n* = 45) sources and strains that were kindly provided by ABC Research, Inc., Dr. Mickie Parish and Margaret Richards, as described in Supplemental Table 1 (available online at doi:10.1155/2011/461321). All strains were stored at −80°C in Luria Bertani NaCl (1%) broth (LBN) and 50% glycerol and subcultured on LBN agar (LA) for genetic characterization.

### 2.2. Sampling Protocol

Suwannee River surface water samples were collected monthly from January to June 2003. Sampling locations included public access sites at the Big Shoals State Park, FL (BP), Stephen Foster State Folk Culture Center in White Springs, FL (WS), Spirit of Suwannee Campground boat ramp (SP), and the Florida Sheriffs Boys Ranch boat ramp (BR). Samples were collected in sterile, glass containers by skimming the surface water and transported in coolers on ice to the laboratory, stored for no more than 24 hours at 4°C, and processed as described below. All media were from Difco Scientific, Inc. unless otherwise specified.

### 2.3. Isolation and Enumeration of *Salmonella*

*Salmonella* water samples (500, 100, 50, 10, and 1 mL) were used to determine most probable number (MPN) by addition to equal volumes of sterile 1% buffered peptone water (BPW) at 2x or 1x (for 1 mL samples) concentration in triplicate [30]. Broth cultures were incubated at 37°C overnight with shaking (New Brunswick Scientific Incubator). These cultures (1 mL of each) were subsequently transferred into 9 mL of Tetrathionate Broth (TT) for selective enrichment at 37°C overnight with shaking. TT broth cultures were then streaked for isolation onto XLD (Oxoid) agar plates, spotted onto LA plates, and incubated at 37°C overnight shaking (Fisher Scientific Incubator). *Salmonella-*positive samples were confirmed by species-specific PCR or DNA probe identification of presumptive positive colonies in XLD and LA, as described below. All confirmed isolates were frozen as mentioned above and stored at –80°C. The MPN/100 mL of each sample from the Suwannee River was determined by the number of replicate enrichment cultures for each dilution that was confirmed by species-specific identification of presumptive positive colonies, as described below.

### 2.4. Species Confirmation

All samples with typical colony morphology for *Salmonella* on XLD agar plates were confirmed by DNA probe colony hybridization and by PCR. For DNA probe assay, colonies were grown overnight on LA at 37°C and transferred by overlay to filter paper (Whatman no. 541), as previously described [31]. Briefly, colonies on filters were lysed in 0.5 M NaOH and 1.5 M NaCl solutions, neutralized in 2 M ammonium acetate (Fisher, Pittsburgh, Pa), and washed in 1x SSC buffer (0.003 M sodium citrate and 0.03 M NaCl). Filters were treated in proteinase K (20 *μ*g/mL) in SSC and washed in SSC. Filters were hybridized at 56°C in buffer with an alkaline-phosphatase-labeled (DNA Technologies, Denmark) DNA probe (5′>CTGGTTGATTTCCTGATCGC>3′) derived from the *S. enterica invA* gene. Filters were washed with 1x SSC at 56°C, followed by washes at room temperature to remove unbound probe, and developed in the presence of NBT/BCIP substrate (Fisher, Pittsburgh, Pa).

For PCR confirmation, broth cultures (1 mL) were extracted by boiling cells from a suspension of one colony in 400 *μ*l of PBS and incubated for 7 min at 100°C. Samples were centrifuged at 13,000 rpm for 3 min, and the supernatant was transferred to a new microcentrifuge tube. Extracted DNA in the supernatant (2 *μ*l) was combined with 23 *μ*l of master mix (Eppendorf), forward primer INVE (5′-TGCCTACAAGCATGAAATGG-3′), and reverse primer INVA (5′-AAACTGGACCACGGTGACAA-3′) for PCR amplification on a Mastercycler Gradient Thermal Cycler (Eppendorf). The following conditions were used: initial denaturation for 3 min. at 94°C, followed by 30 cycles of 1 min. at 94°C, 1 min. at 56°C, and 1 min. at 72°C, with the final extension of 15 min. at 72°C. Samples were combined with 2 *μ*l of 6x loading dye (Promega) and run on 1% agarose gels for visualization of PCR products by ethidium bromide staining. Positive *Salmonella* bands had a length of 457 base pairs. 

### 2.5. Rep-PCR Analysis of *S. enterica* Isolates

DiversiLab Rep-PCR analysis of *S. enterica *isolates was performed according to manufacturer's specifications for the *Salmonella* (BioMerieux). Briefly, genomic DNA from isolates grown on LA plates was extracted using UltraClean Microbial DNA Isolation Kit (Mo Bio Laboratories, Inc.). DNA concentration was determined by spectrometry (SPECTRA max Plus 384, Molecular Devices, Sunnyvale, Calif) and stored at −20°C. PCR amplification used *S. enterica* rep-PCR DNA Fingerprinting Kit (BioMerieux) with AmpliTaq DNA polymerase and GeneAmp 10x PCR Buffer I with Mg Cl_2_ (Applied Biosystems, Inc.). Each PCR reaction (25 *μ*L) included 2 *μ*L of the *S. enterica* primer mix, 0.5 *μ*L of the AmpliTaq DNA polymerase, 2.5 *μ*L of the Buffer I, 18 *μ*L of the Master mix, and approximately 2 *μ*L (50 ng/*μ*L) of template DNA. Amplification was performed in a Mastercycler Gradient Thermal Cycler (Eppendorf) according to the conditions indicated in the *S. enterica* rep-PCR Fingerprinting kit. All rep-PCR amplicons were screened by electrophoresis in a 1.5% agarose gel (Fisher Scientific) in Tris-acetate-EDTA buffer containing ethidium bromide. All amplification products were stored at −20°C. Sample analysis was performed using amplicons (1 *μ*L) loaded onto the DNA microfluidics Labchip and for capillary electrophoresis by the Agilent 2100. Electrophoretograms were analyzed by DiversiLab system software (BioMeriuex) for strain comparison of the DNA similarity to the DiversiLab rep-PCR online library for *S. enterica *(*n* = 352).

### 2.6. Statistical Analysis

The Chi-square test was used to compare segregation of rep-PCR genogroups with strain origin (clinical versus environmental) and serovar [32].

## 3. Results

### 3.1. Recovery of *S. enterica* from Suwannee River

**Figure 1:**
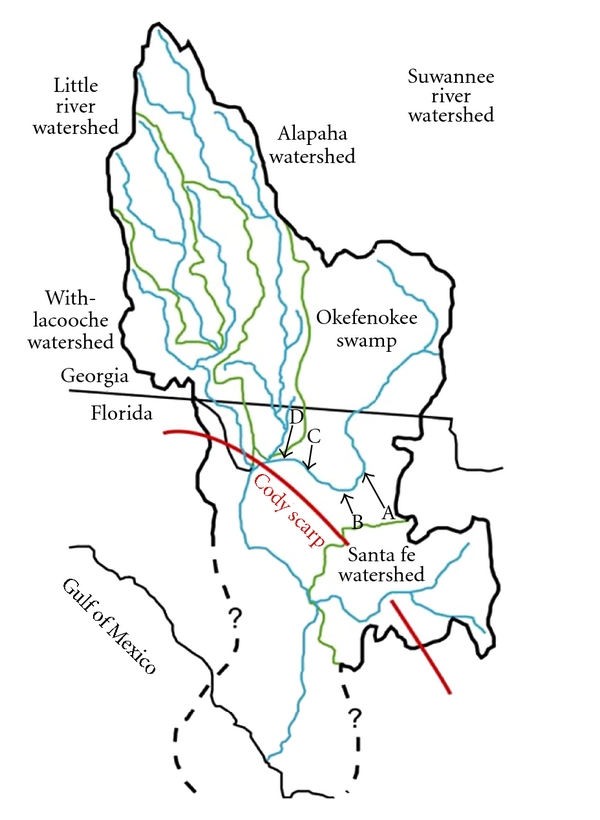
Sampling sites for the Suwannee River. The location of sites on the Suwannee River is shown in relationship to the Suwannee River watershed. Primary sampling locations (arrows) included four sites on the Suwannee River from (A) Big Shoals State Park, (B) White Springs State Park, (C) Spirit of the Suwannee Campground, and (D) Boys Ranch.

*Salmonella* was isolated from the surface water at all sites that were sampled on the Suwannee River (Figure 1). These sites include the Big Shoals State Park (BS), Stephen Foster State Folk Culture Center in White Springs, FL (WS), Spirit of Suwannee Campground (SP), and Florida Sheriffs Boy's Ranch (BR) as shown in Figure 1. Big Shoals has the closest proximity to the source of the river in the Okeefenokee Swamp, while the other downstream sites were in closer proximity to higher-density human activity. We found that 96% of samples were positive for *S. enterica *as indicated by recovery of isolates with the *invA *gene.

Although statistics were not possible because MPN was not performed from replicate samples from each site, the results did show that *Salmonella *levels ranged from not detectable to 5400 MPN/100 mL (Table 1). Interestingly, the two upstream sites, Big Shoals and White Springs, consistently maintained about 10- to 100-fold lower MPN levels January through April, compared to downstream sites, which were from more densely populated regions or located adjacent to agricultural activity. With the exception of the sample collected at White Springs in January, at least 30 isolates were recovered from each time point from all sites and were stored as frozen stocks for additional testing. Total heterotrophic aerobic bacterial counts on LA from Suwannee River water samples during this time period ranged between 1.0 × 10^1^ and 6.9 × 10^2^ CFU mL^−1^ (data not shown).

### 3.2. Genetic Diversity of *S. enterica* Strains from the Suwannee River

**Figure 2:**
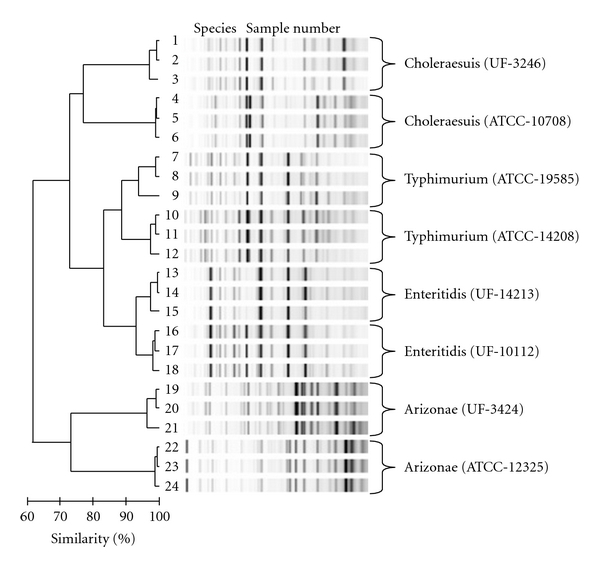
DiversiLab rep-PCR dendrogram. Representative strains are shown to demonstrate the segregation of different serotypes by rep-PCR. Cluster A was comprised of duplicate serovars of subspecies I (Typhimurium, Choleraesuis, and Enteritidis), while cluster B included duplicate serovars of subspecies III (Arizonae). Within each individual serovar, analyzed in triplicates, >94% DNA similarity was observed. More than 70% DNA relatedness was noted between serovars of the same serovars. The scale at the bottom indicates the percentage similarity among fingerprints of each genotype.

The genetic relatedness among isolates of *S. enterica *recovered from the Suwannee River was evaluated by the DiversiLab rep-PCR system. These isolates were compared to other *S. enterica *strains from both clinical and environmental sources and to the DiversiLab *S. enterica *library. This method divides *S. enterica *strains into two main clusters that segregate subspecies III from subspecies I strains (Figure 2). Replicate analysis (*n* = 10) of the same strains (*n* = 6) generally showed at least 95% similarity (data not shown). Therefore, isolates with >95% similarity were considered to be clonal by this assay. A total of 499 strains were examined by rep-PCR, and all strains were >60% similar by this assay. Strains segregated into 16 genogroups using the criteria of >85% DNA similarity for more than two strains, while 14 strains were ungrouped (Supplemental Figure 1S).

Overall, *S. enterica *strains recovered from the Suwannee River were quite diverse and were distributed among 10 genogroups, while 6 isolates did not cluster with other strains (Supplemental Figure 1S). None of the Suwannee isolates clustered with subspecies 3 (Arizonae). Significant (*P* < 0.001) genetic relatedness was observed among the Suwannee River isolates by rep-PCR, and these strains were generally distinct from strains derived from other sources. Most (74%) of the Suwannee River strains segregated into only two genogroups, namely, 10 and 15. Each of these groups contained only one isolate from non-Suwannee sources, including one from a clinical case and another from a tomato. Conversely, 69% of strains from the DiversiLab Library, consisting of isolates from clinical sources, were distributed into genogroups 5, 6, 11, and 13, while only 12% of Suwannee River isolates were included in any of these groups. Interestingly, Suwannee River isolates were also distinct from strains derived from other environmental sources including those from central Florida, such as frogs, oranges, or lakes (Table 2). For example, the isolates from central Florida lakes clustered into genogroups 5 and 11 that were predominantly populated with strains of clinical origin.

Examination of the distribution of Suwannee River isolates by sampling site showed that the different genogroups were found among various sampling locations, and the genogroups that were most commonly populated with Suwannee isolates (10 and 15) were found at all sites (Table 3). Similarly, individual genogroups were evenly distributed by month (Table 4). Although strains were isolated from enriched samples, clonal isolates (>95% similarity) did not necessarily correspond to the same sample site or time. However, because of the low number and unequal distribution of isolates collected from each site or time point, statistical analysis was not performed with respect to sample site or seasonality, and no clear relationship was established.

### 3.3. Distribution of *Salmonella* Serotypes in Relationship to DiversiLab Genogroups

*S. enterica *isolates (*n* = 30) recovered from the Suwannee River in a prior study in 1998-1999 [33] were serotyped, and serotypes included Inverness (*n* = 9), Muenchen (*n* = 3), Rubislaw (*n* = 6), Braenderup (*n* = 2), and one strain each of Montevideo, Newport, Johannesburg, and Cubana. Some strains were untypeable strains (*n* = 3) or rough (*n* = 3). Specific serotypes and source of strains are shown in Table 5. Rep-PCR analysis showed agreement with serology of *S. enterica* derived from the Suwannee River and elsewhere, and strains (70%) with the same serovar were frequently clonal (>95% similar) with one or more strains from independent sources. However, rep-PCR also revealed genetic distinctions among strains of the same serovar, as serotypes frequently segregated into multiple genogroups (Table 6). Furthermore, the genetic relatedness of isolates that were un-typeable by serology was revealed by rep-PCR. For example, an un-typeable Suwannee River isolate (UF-14) was not identified as *S. enterica *by serotyping but demonstrated 96% DNA similarity to other Suwannee River isolates that were serotyped as Braenderup (UF-8 and UF-9) in genogroup 9 (Supplemental Figure 1(S)). Similarly, a rough *S. enterica* strain (UF-29) demonstrated >95% DNA similarity with two Suwannee River isolates serotyped as Inverness (UF-28 and UF-30) within genogroup 10. A high level of DNA similarity (>90%) was also noted among several Suwannee River isolates with serotype Rubislaw and another un-typeable strain (UF-3) within genogroup15. However, two un-typeable (UF-12, UF-15) strains did not cluster with any other isolates at the 85% level of DNA similarity. A single clinical *S. enterica* Inverness serotype clustered with 39 Suwannee River isolates in genogroup 10, including 8 Suwannee River isolates that were also Inverness serotype. The remaining Suwannee River isolate that serotyped as Inverness was found in genogroup 5 that also included Suwannee River isolates which serotyped as Muenchen and Johannesburg. Several of the Suwannee River strains indicated very high levels (≥90%) of DNA relatedness to important clinical serovars such as Typhimurium, Montevideo, and Muenchen.

## 4. Discussion

*S. enterica *was widely distributed in the upper Suwannee River and was recovered from multiple locations at different points of time. Serotyping and genetic analyses illustrated the diversity of these strains but also showed that some genotypes may be more prevalent in the Suwannee River than others, as >70% of strains clustered into only two genogroups. Furthermore, these genogroups were distinct as most strains derived from clinical and other environmental sources were excluded. Interestingly, strains recovered from central Florida lakes in a preliminary study using similar methodology were much more likely to cluster with strains from clinical sources. Although the number of strains in this study is too small to draw conclusions about these populations, results suggest that genotypes of *S. enterica *may be differentially distributed in Florida aquifers.

The DiversiLab rep-PCR assay showed some agreement with serotyping, as serotypes from different sources frequently clustered together. However, genetic typing also indicated that some stains with the same serotype were genetically diverse. For example, strains from the Suwannee River with serotype Inverness were mostly (8 of 9) in genogroup 10, which also included a clinical Inverness strain, but one of the Suwannee River Inverness isolates diverged to genogroup 5 and was more genetically related to strains with serotype Muenchen. Prior descriptions of rep-PCR typing have also shown mixed results. Weigel et al. [29] noted greater discrimination power for rep-PCR compared to PFGE, but Kerouanton et al. [34] found it less discriminatory than either ribotyping or AP-PCR. Chmielewski et al. [35] found REP-PCR and ERIC-PCR to be highly discriminatory among isolates from Poland, but reproducibility was an issue with band number and positioning varying with the model of the thermocycler [36]. The Diversilab system (bioMerieux) for rep-PCR used in the present study is distinctive from other rep-PCR assays due to the standardized reagents, online database, internal controls for peak size and height, and the use of capillary electrophoresis for characterization of PCR products. We found that independent samples of the same *Salmonella* strain were highly reproducible and always clustered with >95% similarity. Wise et al. [27] recently found this system was an excellent predictor of serotype, and Kilic et al. [28] found it was a “reasonable alternative to PFGE.”

The top 10 disease-associated serotypes in 2009 included Newport, Typhimurium, Javiana, Muenchen, Heidelberg, Montevideo, Oranienburg, and St. Paul. While the true diversity of *Salmonella* serovars in the Florida aquifers is still unknown, the present study identified 8 serovars (Inverness, Muenchen, Rubislaw, Braenderup, Montevideo, Newport, Johannesburg, and Cubana) and 10 genogroups from this region. A prior investigation in 1989 reported 14 *Salmonella* serovars derived from the Suwannee River that included Allandale, Aqua, Braenderup, Daytona, Gaminara, Hartford, Inverness, Montevideo, Muenchen, Paratyphi B, Saintpaul, subspecies IV, Tallahassee, and Typhimurium [37]. Another study in 1997 reported 7 serovars (Rubislaw, Tallahassee, Gaminara, Javiana, Muenchen, Inverness, and Hartford), and in 1998, 10 serovars were reported (Allandale, Montevideo, Gaminara, Hartford, Inverness, Javiana, Muenchen, Rubislaw, Tallahassee, and Arizonae) by the Florida Department of Agriculture Consumer Services [38]. Thus, Muenchen and Inverness were common to all studies (including the present study), while Braenderup, Montevideo, and Rubislaw were found in more than one study. Newport, Johannesburg, and Cubana were unique to the present study.

The present study conducted in north Florida showed some similarity to a recent survey of *Salmonella *from the Little River in southern Georgia portion of the upper Suwannee River watershed [17]. Both reports identified Muenchen and Rubislaw among the predominant serotypes, but 40.6% of strains in the prior study were identified as *S. enterica *subsp. Arizonae, which was not recovered from the Suwannee River either by serotype or rep-PCR genotype. They concluded that recovery of Muenchen in the watershed was epidemiologically significant, as its incidence is increasing in human cases in Georgia (34% increase over 10 years). Furthermore, the Muenchen strains from the Suwannee River were associated with a predominantly clinical (genogroup 5) genotype. Recent *Salmonella *outbreaks in Florida have identified Braenderup [39], Javiana [40], St. Paul [41]. No Javiana was included in this study, and the Braenderup identified from the Suwannee River formed a unique rep-PCR cluster (genogroup 9). Neither study identified the most common serotype-associated salmonellosis, namely, serotype Enteritidis, from water samples [1]. Furthermore the rep-PCR Genogroup 6 that was identified with clinical Enteritidis strains did not contain any isolates from the Suwannee River. However, it should be noted the genogroup 7 that included clinical Typhimurium strains did cluster with 4 isolates from the Suwannee River. Thus, perhaps the most intriguing finding of the present study was the identification of *Salmonella* strains in the Suwannee River that were genetically associated with strains from clinical sources.

Although these investigations are not a systematic or exhaustive survey of Suwannee River watershed, they demonstrate the diversity of *Salmonella *in possible irrigation sources and suggest the presence of potential pathogens. Franz and Van Bruggen [13] reported that the prevalence of pathogens in the environment is inversely proportional to the genetic diversity of the biome and that eutrophic environments promote decreased diversity. While the present investigation did not examine environmental parameters with respect to distribution of *Salmonella* in Florida, there are striking differences in the human population densities associated with upstream versus downstream sites. The upstream sites are in closer proximity to the source of this river and greater distance from agriculture and other human impact. For example, the most upstream site at Big Shoals is in a state park and is essentially unpopulated with no agriculture in close proximity to the river, while the other sites are in direct contact with human populations. These differences may explain the 10- to 100-fold increase in the MPN mL^−2^ at downstream sites relative to upstream. However, there were no striking differences observed in terms of diversity of *Salmonella *from the different sites on Suwannee River. Future studies are needed to examine the complex environmental parameters, especially in relationship to nutrient availability, agricultural input, wildlife distribution, flow rates, rainfall and other factors, that may impact the microbial diversity and survival of *Salmonella*.

## Supplementary Material

Genogroups for *Salmonella enterica* strains were based on DiversiLab rep-PCR and included Suwannee River isolates (*n*=110) from this study, which were compared to other environmental (*n*=47) or clinical (*n*=28) strains and to an online DiversiLab library (*n*=314). Analysis of 499 environmental and clinical *Salmonella* Isolates was assembled into a dendrogram based on rep-PCR fingerprinting profiles at ≥85% DNA similarity level, using DiversiLab software. Genogroups (*n*=16) were comprised of clusters with >2 strains each. *Salmonella* isolates from the Suwannee River clustered into 10 genogroups, but the majority of strains were found Genogroups 10 and 15.
Legend for Figure 1S should be changed to read “*S. enterica* genogroups were derived from DiversiLab rep-PCR as described in the Materials and Methods. Strains included Suwannee River isolates from this study (highlighted), which were compared to other environmental or clinical strains. Dendrogram was assembled from rep-PCR fingerprinting profiles at ≥85% DNA similarity level, using DiversiLab software. The strains comprised a total of 16 genogroups with >2 strains each. Gel-like images, serovar identity, isolation source, and identification number for each *Salmonella* isolate are provided. Abbreviations are used for descriptions of sampling locations on the Suwannee River and include: BS (Big Shoals), WS (White Springs), SP (Spirit of Suwannee), and BR (Boy's Ranch). Additional strains from our collection were from ATCC or provided by Dr. Parish, and strain source is indicated when known. DiversiLab strains are designated by SE number.”

## Figures and Tables

**Table 1:** Recovery of *Salmonella* from the Suwannee River.

Site^a^	*Salmonella* recovered^b^ (MPN/100 mL)			
	January	February	March	April
Spirit of Suwannee	220	5400	4500	1100
Boys Ranch	210	5400	230	1300
White Springs	<18	700	460	790
Big Shoals	78	240	790	45

^a^Sites on the Suwannee River are described in Table 1.

^b^MPN enumeration is described in the Materials and Methods.

**Table 2:** Distribution of rep-PCR genogroups related to the source of *Salmonella* isolates.

Rep-PCCR genogroup^a^	Number of isolates by source					
	Total	DiversiLab	ATCC	Suwannee River	FL lakes	Other (source)
1	4	1	3	0	0	0
2	5	5	0	0	0	0
3	4	0	0	3	0	1 (Orange)
4	3	0	2	0	0	1 (Orange)
5	187	161	4	9	4	6 (Orange)
						2 (Cantaloupe)
						1 (Toad)
6	42	35	7	0	0	0
7	24	10	7	3	0	4 (Orange)
8	7	0	1	2	1	3 (Orange)
9	3	0	0	3	0	0
10	40	0	1	39	0	0
11	67	51	0	3	13	0
12	15	13	0	2	0	0
13	21	20	0	0	0	1 (Alfalfa)
14	15	4	2	5	0	4 (Frog)
15	36	0	0	35	0	1 (Tomato)
16	5	4	0	0	0	1 (Reptile
N.C.^b^	21	10	1	6	1	2 (Water/soil)
						1 (Reptile)

Total	499	314	28	110	19	28

^a^Strains were clustered into genogroups with more than two strains showing >85% similarity between strains.

^b^N.C.: Strains that did not cluster and showed <85% to other strains.

**Table 3:** Distribution of *S. enterica *genogroups by location for strains recovered from the Suwannee River.

Genogroup^a^	Sampling sites^b^					
	Big Shoals	White Springs	Spirit of Suwannee	Boy's Ranch	Other	Total
3	—	—	3	—	—	3
5	—	2	2	2	3	9
7	2	1	—	—	—	3
8	—	2	—	—	—	2
9	—	2	1	—	—	3
10	4	3	14	11	7	39
11	—	—	1	2	—	3
12	1	—	—	—	1	2
14	1	—	3	—	1	5
15	9	7	8	3	8	35

Total (%)	17 (16.3%)	17 (16.3%)	32 (30.8%)	18 (17.3%)	20 (19.2%)	104 (100.0%)

^a^Genogroups are described in Figure 1(S) as determined by DiversiLab rep-PCR and described in Materials and Methods.

^b^Sampling sites are described in text except for “others” which were collected in 1999-1998 and were downstream of sampling sites in this study.

**Table 4:** Distribution of *S. enterica *genogroups by month for strains recovered from the Suwannee River.

Genogroup^a^	Jan	Feb	March	April	May	June	Total
3	3	—	—	—	—	—	3
5	—	3	—	1	—	—	4
7	—	1	—	—	2	—	3
8	—	—	—	—	2	—	2
10	10	2	5	4	4	5	30
11	—	1	2	—	—	—	3
11	—	1	—	—	—	—	1
12	1	—	—	—	—	3	4
15	9	3	5	2	5	3	27

Total %	23 (29.9%)	11 (14.3%)	12 (15.6%)	7 (9.1%)	13 (16.8%)	11 (14.3%)	77 (100%)

^a^Genogroups are described in Figure 1(S) as determined by DiversiLab rep-PCR and described in Materials and Methods.

**Table 5:** Detailed serology of *S. enterica *isolated from Suwannee River water.

Serotype^a^ (number of isolates)	Strain designation (Genogroup)	Serovar^b^	Antigenic Structure^c^
Braenderup (2)	UF-8; UF-9	C1	6,7:e,h:e,n,z_15_
Cubana (1)	UF-18	G2	13,23:z_29_:-
Inverness (9)	UF-5; UF-13; UF-17; UF-20; UF-22; UF-26; UF-27; UF-28; UF-30	P	38:k:1,6
Johannesburg (1)	UF-7	R	1,40:b:e,n,x
Montevideo (1)	UF-10	C1	6,7:g,m,s:-
Muenchen (3)	UF-16; UF-24; UF-19	C2	6,8:d:1,2
Newport (1)	UF-6	C2	6,8:e,h:1,2
Rubislaw (6)	UF-1; UF-2; UF-4; UF-21; UF-23; UF-25	F	11:r:e,n,x
Un-typeable (6)	UF-3; UF-11; UF-12; UF-14; UF-15; UF-29	—	—

^a^Serology for *invA* probe-positive strains (*n* = 30) was determined by the *Salmonella* Reference Center, Philadelphia, PA.

^b^Serovars reflect subspecies designation.

^c^Somatic antigen: phase I flagellar antigen and phase II flagella antigen (if present).

**Table 6:** Relationship of serotype to genogroup.

Serotype^a^	Associated genogroups	Source (number of isolates)
Arizonae	16	ATCC (1), DiversiLab (4)
	N.C.^b^	ATCC (1)

Braenderup	9	Suwannee River (2)

Cholerasuis	5	ATCC (2)

Enteritidis	1	ATCC (2)
	6	ATCC (2)
	N.C.	Soil (1)

Gaminara	3	Orange (1)
	4	ATCC (1); Orange (1)
	5	Orange (1)
	6	ATCC (1)

Hartford	5	ATCC (1); Toad (1); Orange (1)

Inverness	5	Suwannee River (1)
	10	Suwannee River (8); ATCC (1)

Montevideo	15	ATCC (1); Suwannee River (1)

Muenchen	5	Suwannee River (3)
	8	Unknown (1)

Newport	6	ATCC (1)
	14	Suwannee River (1); Frog (4)

Rubislaw	1	ATCC (1)
	15	Suwannee River (6)

St. Paul	7	Orange (7)

Typhimurium	6	ATCC (1)
	7	ATCC (3)
	11	DiversiLab (4)

Un-typeable	9	Suwannee River (1)
	10	Suwannee River (1)
	12	Suwannee River (1)
	15	Suwannee River (1)
	N.C.	Suwannee River (2)

^a^Serotypes with at least two representative isolates are shown with respect to DiversiLab rep-PCR genogroups described in Supplemental Figure 1(S).

^b^N.C.: not clustered. Strains that did not show >85% similarity to at least 2 other strains were not designated as belonging to a genogroup.

## References

[1] CDC (2010). Preliminary FoodNet Data on the incidence of infection with pathogens transmitted commonly through food—10 states, 2009. *Morbidity and Mortality Weekly Report*.

[2] Mead PS, Slutsker L, Dietz V (1999). Food-related illness and death in the United States. *Emerging Infectious Diseases*.

[3] Stevens MP, Humphrey TJ, Maskell DJ (2009). Molecular insights into farm animal and zoonotic salmonella infections. *Philosophical Transactions of the Royal Society B*.

[4] Santamaría J, Toranzos GA (2003). Enteric pathogens and soil: a short review. *International Microbiology*.

[5] Birkhead GS, Morse DL, Levine WC (1993). Typhoid fever at a resort hotel in New York: a large outbreak with an unusual vehicle. *Journal of Infectious Diseases*.

[6] Cook KA, Dobbs TE, Hlady WG (1998). Outbreak of Salmonella serotype Hartford infections associated with unpasteurized orange juice. *Journal of the American Medical Association*.

[7] Parish ME (1998). Coliforms, Escherichia coli and Salmonella serovars associated with a citrus-processing facility implicated in a salmonellosis outbreak. *Journal of Food Protection*.

[8] Krause G, Terzagian R, Hammond R (2001). Outbreak of Salmonella serotype anatum infection associated with unpasteurized orange juice. *Southern Medical Journal*.

[9] Harris LJ, Farber JN, Beuchat LR (2003). Outbreaks associated with fresh produce: incidence, growth, and survival of pathogens in fresh and fresh-cut produce. *Comprehensive Reviews in Food Science and Food Safety*.

[10] Heaton JC, Jones K (2008). Microbial contamination of fruit and vegetables and the behaviour of enteropathogens in the phyllosphere: a review. *Journal of Applied Microbiology*.

[11] Teplitski M, Barak JD, Schneider KR (2009). Human enteric pathogens in produce: un-answered ecological questions with direct implications for food safety. *Current Opinion in Biotechnology*.

[12] Whipps JM, Hand P, Pink DAC, Bending GD (2008). Human pathogens and the phyllosphere. *Advances in Applied Microbiology*.

[13] Franz E, Van Bruggen AHC (2008). Ecology of E. coli O157:H7 and Salmonella enterica in the primary vegetable production chain. *Critical Reviews in Microbiology*.

[14] Barak JD, Liang AS (2008). Role of soil, crop debris, and a plant pathogen in Salmonella enterica contamination of tomato plants. *PLoS ONE*.

[15] Winfield MD, Groisman EA (2003). Role of nonhost environments in the lifestyles of Salmonella and Escherichia coli. *Applied and Environmental Microbiology*.

[16] Cooley M, Carychao D, Crawford-Miksza L (2007). Incidence and tracking of escherichia coli O157:H7 in a major produce production region in California. *PLoS ONE*.

[17] Haley BJ, Cole DJ, Lipp EK (2009). Distribution, diversity, and seasonality of waterborne salmonellae in a rural watershed. *Applied and Environmental Microbiology*.

[18] Gaertner JP, Garres T, Becker JC, Jimenez ML, Forstner MRJ, Hahn D (2009). Temporal analyses of Salmonellae in a headwater spring ecosystem reveals the effects of precipitation and runoff events. *Journal of Water and Health*.

[19] Klerks MM, Franz E, Van Gent-Pelzer M, Zijlstra C, Van Bruggen AHC (2007). Differential interaction of Salmonella enterica serovars with lettuce cultivars and plant-microbe factors influencing the colonization efficiency. *ISME Journal*.

[20] Guo X, Chen J, Brackett RE, Beuchat LR (2001). Survival of Salmonellae on and in tomato plants from the time of inoculation at flowering and early stages of fruit development through fruit ripening. *Applied and Environmental Microbiology*.

[21] Barak JD, Gorski L, Liang AS, Narm KE (2009). Previously uncharacterized Salmonella enterica genes required for swarming play a role in seedling colonization. *Microbiology*.

[22] Davis MM, Hicks DW Water resources of the upper suwannee river watershed.

[23] CDC (2008). Foodnet facts and figures-number of infections and incidence per 100,000 persons: all sites, by site 2008. *Morbidity and Mortality Weekly Report*.

[24] Anselmo RJ, Barrios H, Viora S (1989). Comparative study of four methods for Salmonella isolation from surface waters. *Revista Argentina de Microbiologia*.

[25] Baudart J, Lemarchand K, Brisabois A, Lebaron P (2000). Diversity of Salmonella strains isolated from the aquatic environment as determined by serotyping and amplification of the ribosomal DNA spacer regions. *Applied and Environmental Microbiology*.

[26] Polo F, Figueras MJ, Inza I, Sala J, Fleisher JM, Guarro J (1999). Prevalence of Salmonella serotypes in environmental waters and their relationships with indicator organisms. *Antonie van Leeuwenhoek*.

[27] Wise MG, Siragusa GR, Plumblee J, Healy M, Cray PJ, Seal BS (2009). Predicting Salmonella enterica serotypes by repetitive sequence-based PCR. *Journal of Microbiological Methods*.

[28] Kilic A, Bedir O, Kocak N (2010). Analysis of an outbreak of Salmonella enteritidis by repetitive-sequence- based PCR and pulsed-field gel electrophoresis. *Internal Medicine*.

[29] Weigel RM, Qiao B, Teferedegne B (2004). Comparison of pulsed field gel electrophoresis and repetitive sequence polymerase chain reaction as genotyping methods for detection of genetic diversity and inferring transmission of Salmonella. *Veterinary Microbiology*.

[30] Andrews WH, Hammack T (2000). Bacteriological Analytical Manual. *Salmonella*.

[31] Wright AC, Miceli GA, Landry WL, Christy JB, Watkins WD, Morris JG (1993). Rapid identification of Vibrio vulnificus on nonselective media with an alkaline phosphatase-labeled oligonucleotide probe. *Applied and Environmental Microbiology*.

[32] Wackerly DD, Mendenhall WI, Scheaffer RL (1996). *Mathematical Statistics with Applications*.

[33] Rajabi M (1999). *Detection and Isolation of Salmonella spp. from the Suwannee River*.

[34] Kerouanton A, Brisabois A, Grout J, Picard B (1996). Molecular epidemiological tools for Salmonella Dublin typing. *FEMS Immunology and Medical Microbiology*.

[35] Chmielewski R, Wieliczko A, Kuczkowski M, Mazurkiewicz M, Ugorski M (2002). Comparison of ITS profiling, REP- and ERIC-PCR of Salmonella Enteritidis isolates from Poland. *Journal of Veterinary Medicine B*.

[36] Johnson JR, Clabots C, Azar M, Boxrud DJ, Besser JM, Thurn JR (2001). Molecular analysis of a hospital cafeteria-associated salmonellosis outbreak using modified repetitive element PCR fingerprinting. *Journal of Clinical Microbiology*.

[37] Glatzer MB, Heil D, Thompson R, Porter W (1990). Special study of incidence of Salmonella in Suwannee Sound, Florida. *Cooperative Study by: Florida Department of Natural Resources FDoAaCS, and U.S. Food and Drug Administration*.

[38] DACS. ECT

[39] Bidol SA, Daly ER, Rickert RE (2007). Multistate outbreaks of Salmonella infections associated with raw tomatoes eaten in restaurants—United States, 2005-2006. *Morbidity and Mortality Weekly Report*.

[40] CDC (2002). Outbreak of Salmonella serotype Javiana infections—Orlando, Florida, June 2002. *Morbidity and Mortality Weekly Report*.

[41] Jain S, Bidol SA, Austin JL (2009). Multistate outbreak of Salmonella Typhimurium and Saintpaul infections associated with unpasteurized orange juice-United States, 2005. *Clinical Infectious Diseases*.

