# Supplementary figures and images for: Distribution and Genetic Diversity of Salmonella enterica in the Upper Suwannee River
Source: Int J Microbiol. 2011 Dec 13;2011:461321. doi: 10.1155/2011/461321 (PMC3278925; doi:10.1155/2011/461321)

## Slide 1
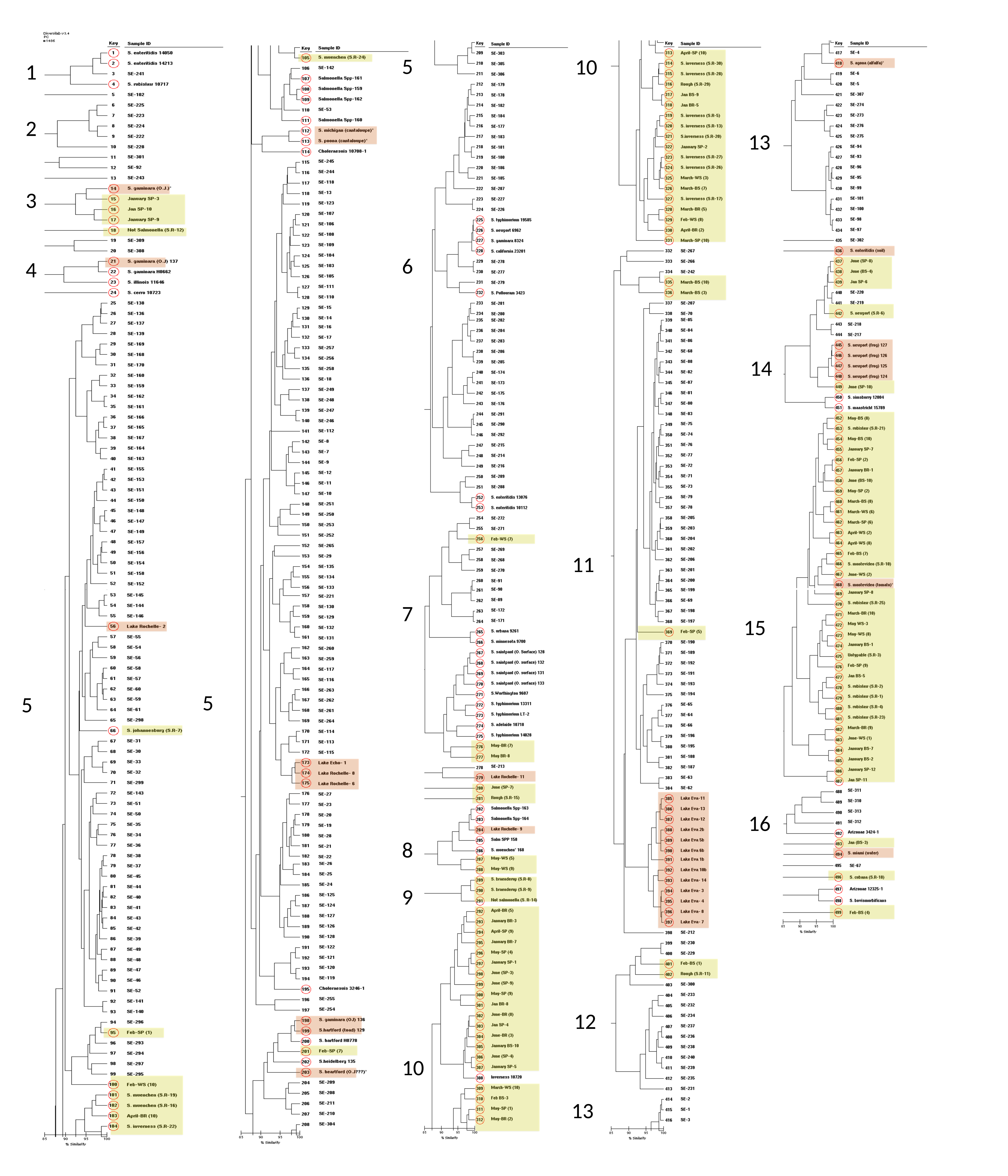

10
11
12
13
14
15
16
6
5
1
2
3
6
4
7
5
5
8
9
10
13

## Slide 2
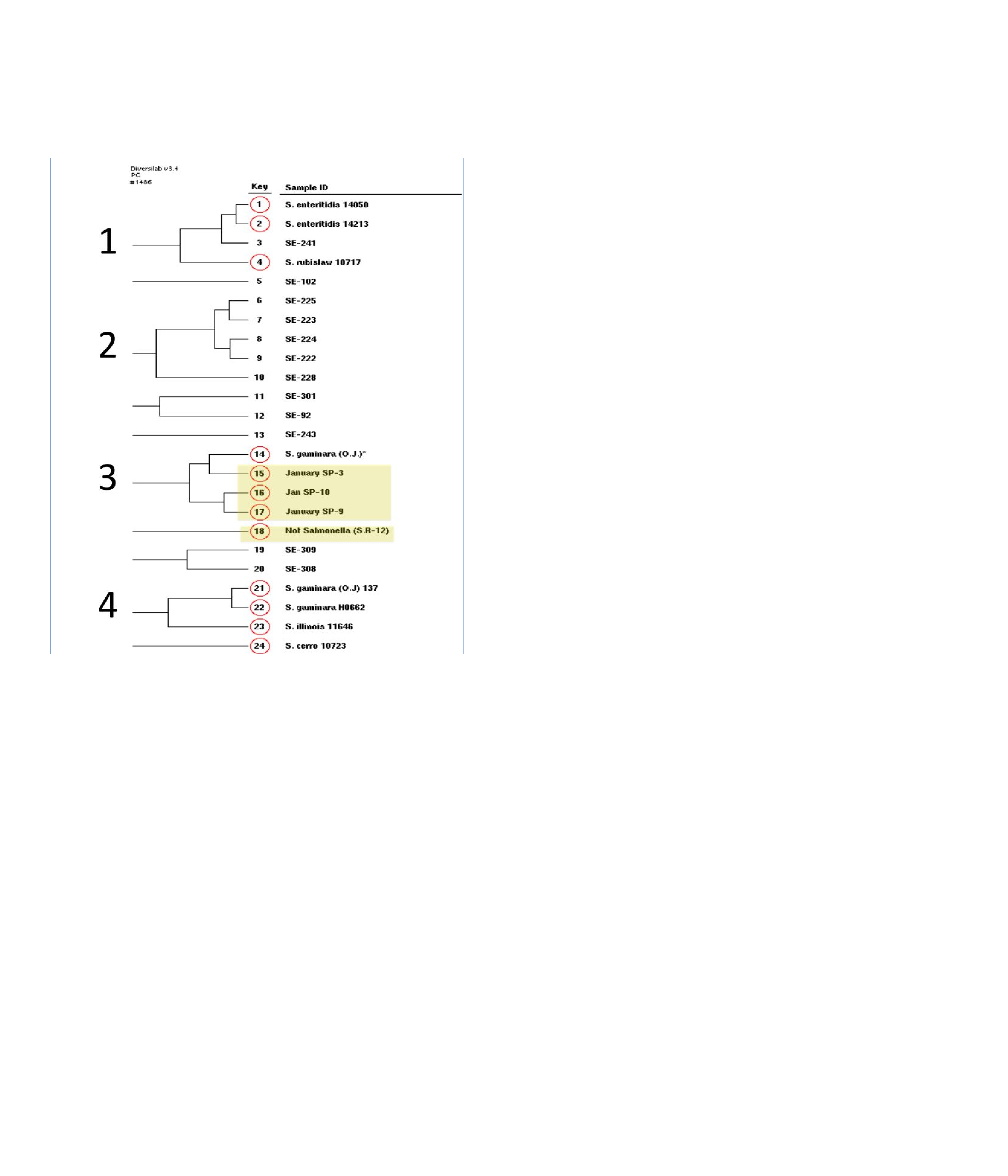

## Slide 3
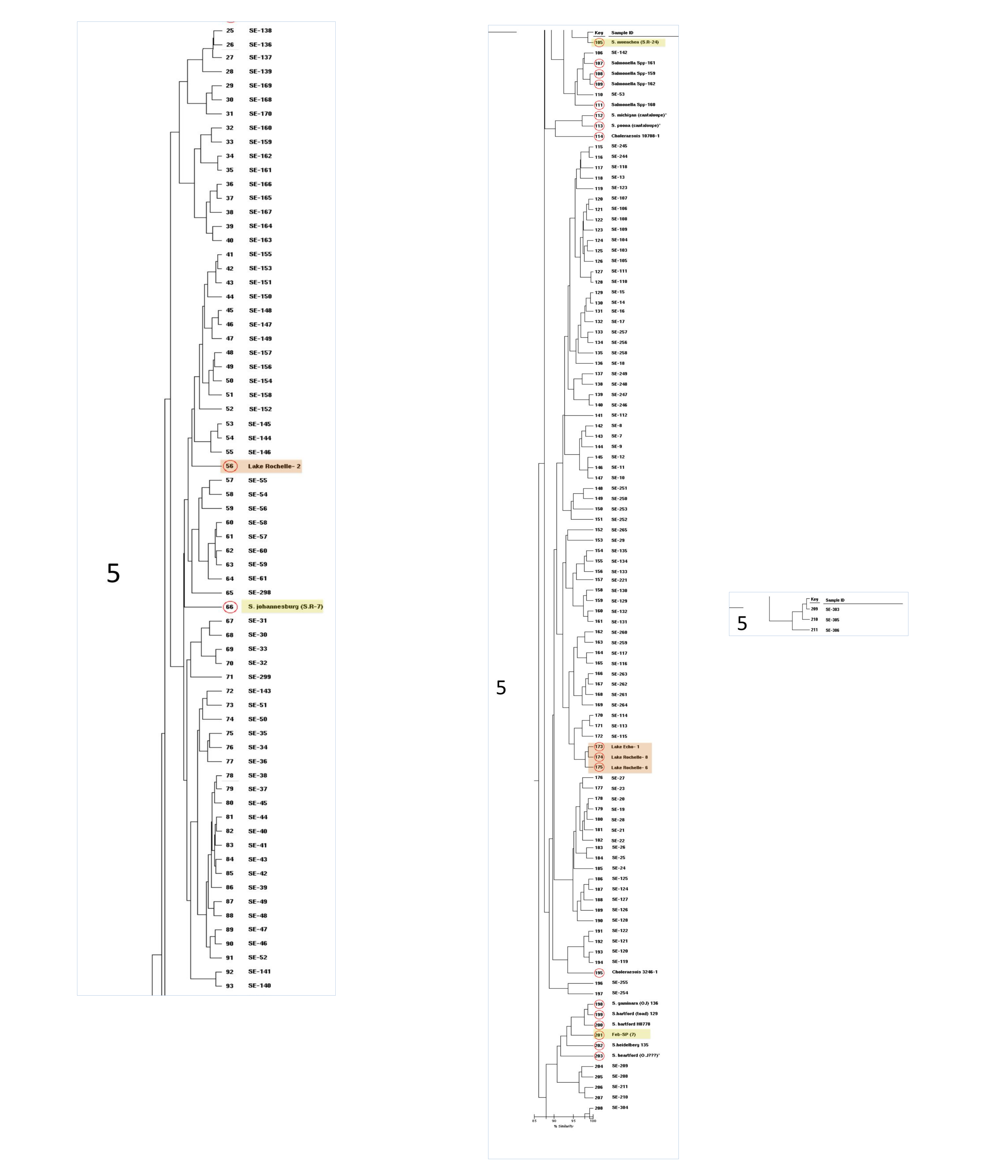

## Slide 4
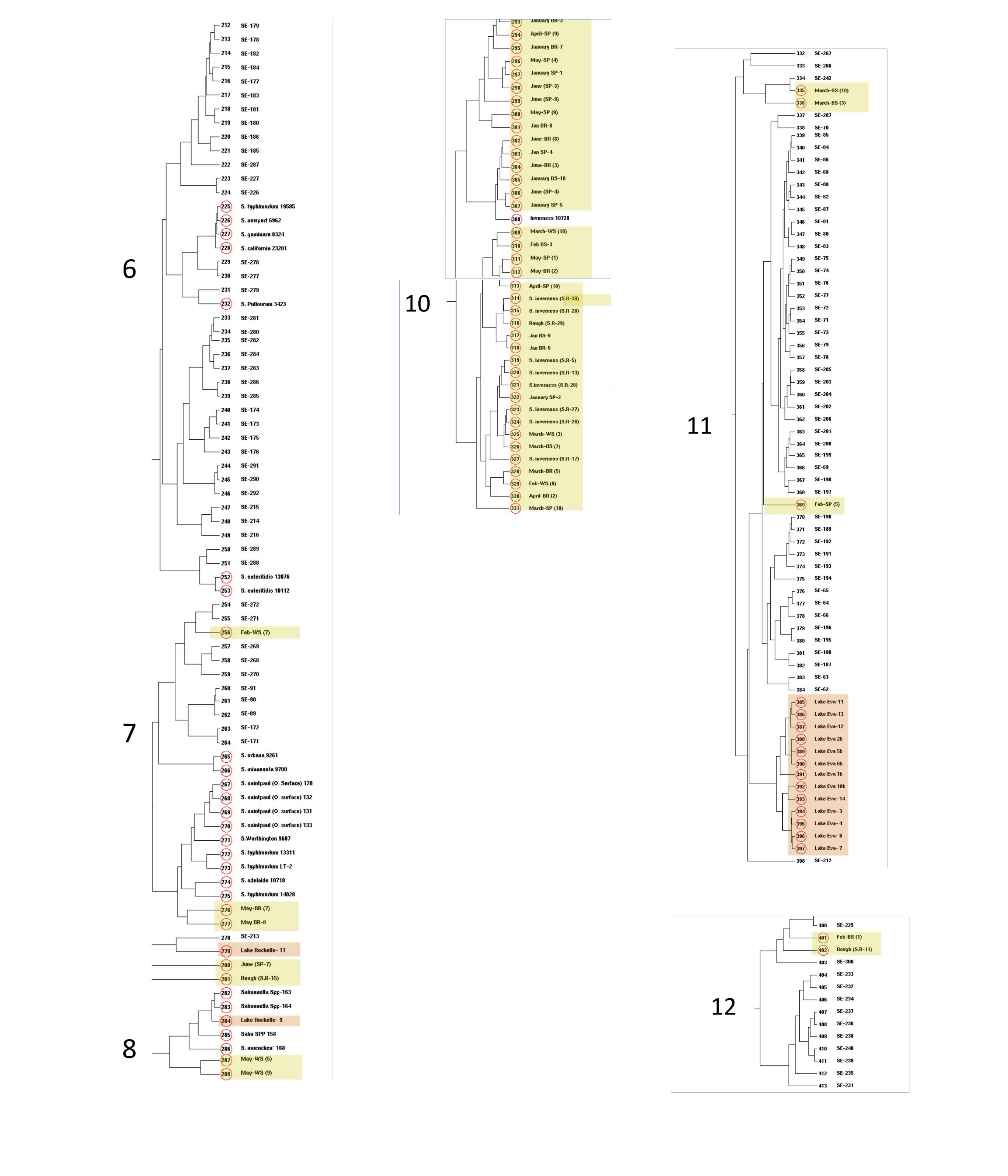

## Slide 5
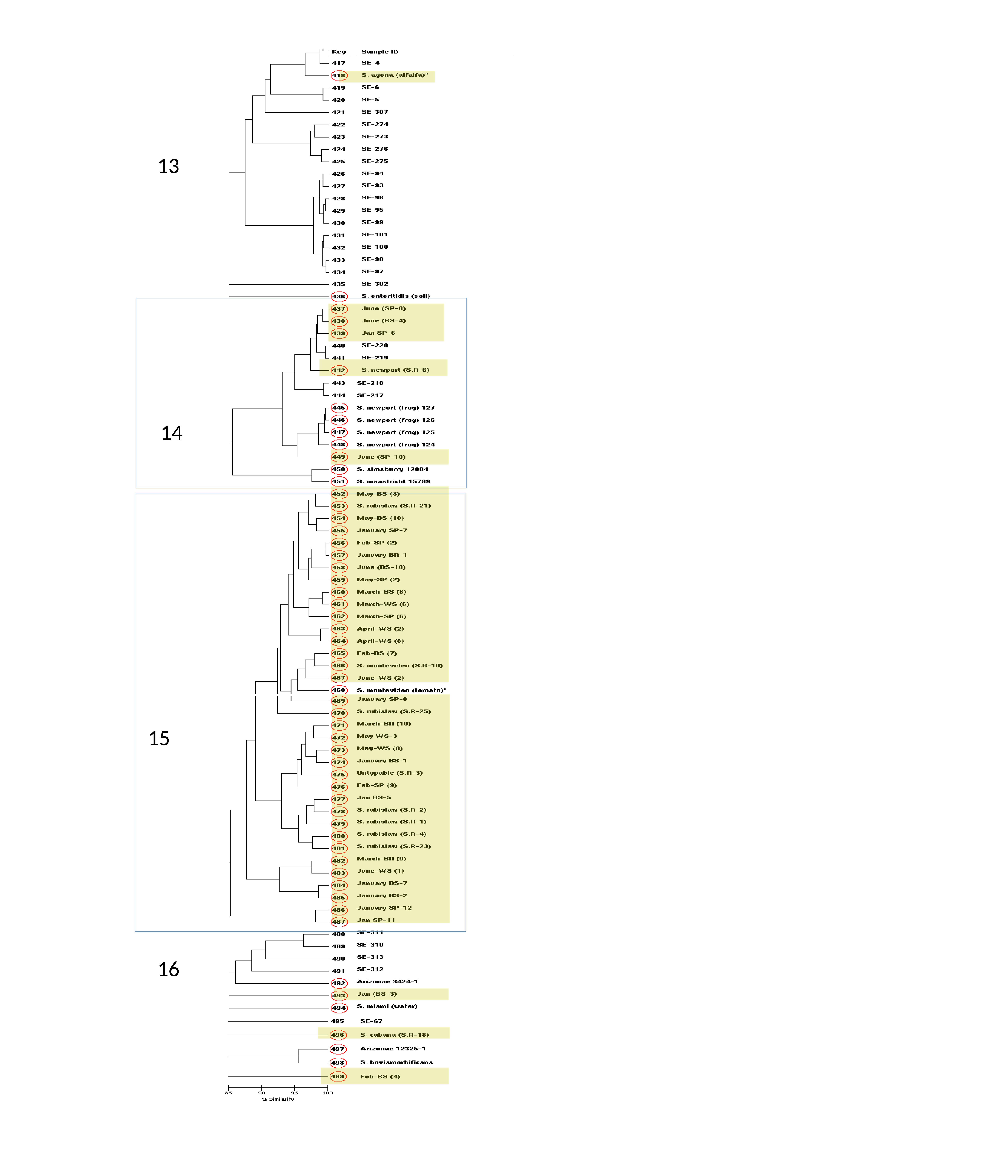

13
14
15
16

Supplement: Supplementary file 1 — Genogroups for Salmonella enterica strains were based on DiversiLab rep-PCR and included Suwannee River isolates (n=110) from this study, which were compared to other environmental (n=47) or clinical (n=28) strains and to an online DiversiLab library (n=314). Analysis of 499 environmental and clinical Salmonella Isolates was assembled into a dendrogram based on rep-PCR fingerprinting profiles at ≥85% DNA similarity level, using DiversiLab software. Genogroups (n=16) were comprised of clusters with >2 strains each. Salmonella isolates from the Suwannee River clustered into 10 genogroups, but the majority of strains were found Genogroups 10 and 15. Legend for Figure 1S should be changed to read “S. enterica genogroups were derived from DiversiLab rep-PCR as described in the Materials and Methods. Strains included Suwannee River isolates from this study (highlighted), which were compared to other environmental or clinical strains. Dendrogram was assembled from rep-PCR fingerprinting profiles at ≥85% DNA similarity level, using DiversiLab software. The strains comprised a total of 16 genogroups with >2 strains each. Gel-like images, serovar identity, isolation source, and identification number for each Salmonella isolate are provided. Abbreviations are used for descriptions of sampling locations on the Suwannee River and include: BS (Big Shoals), WS (White Springs), SP (Spirit of Suwannee), and BR (Boy's Ranch). Additional strains from our collection were from ATCC or provided by Dr. Parish, and strain source is indicated when known. DiversiLab strains are designated by SE number.” [file 461321.f1.zip › 461321.f1.pptx]

## Slide 1
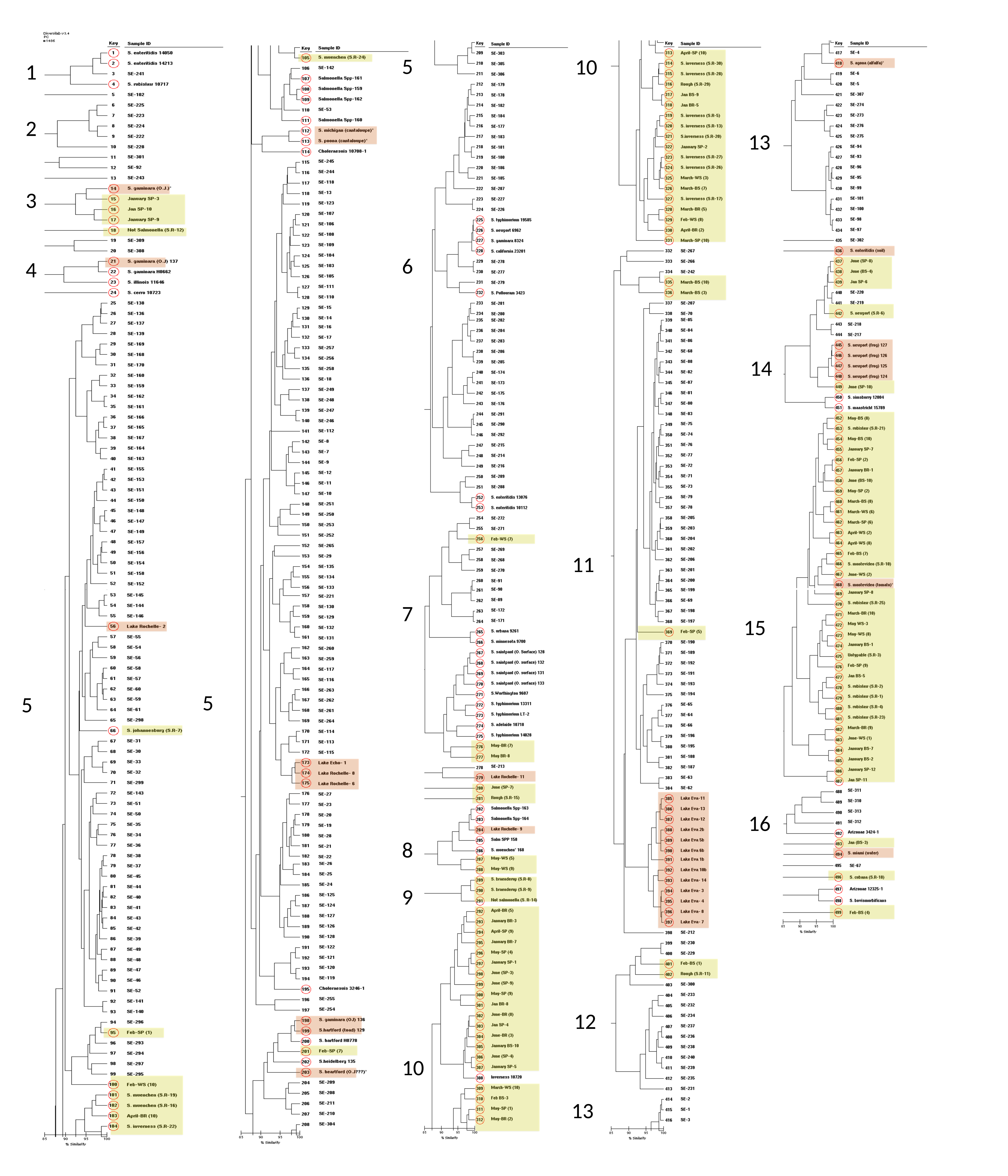

10
11
12
13
14
15
16
6
5
1
2
3
6
4
7
5
5
8
9
10
13

## Slide 2
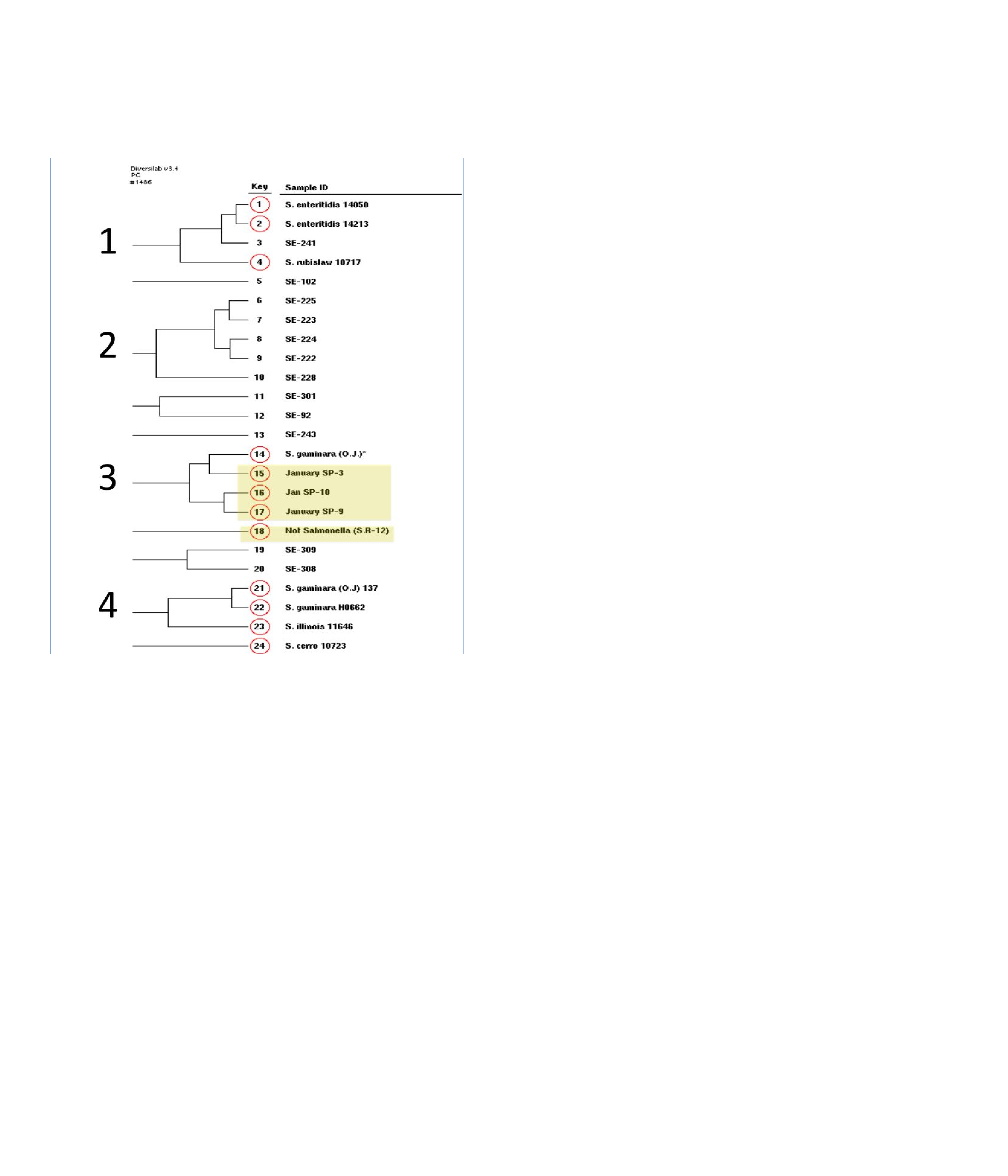

## Slide 3
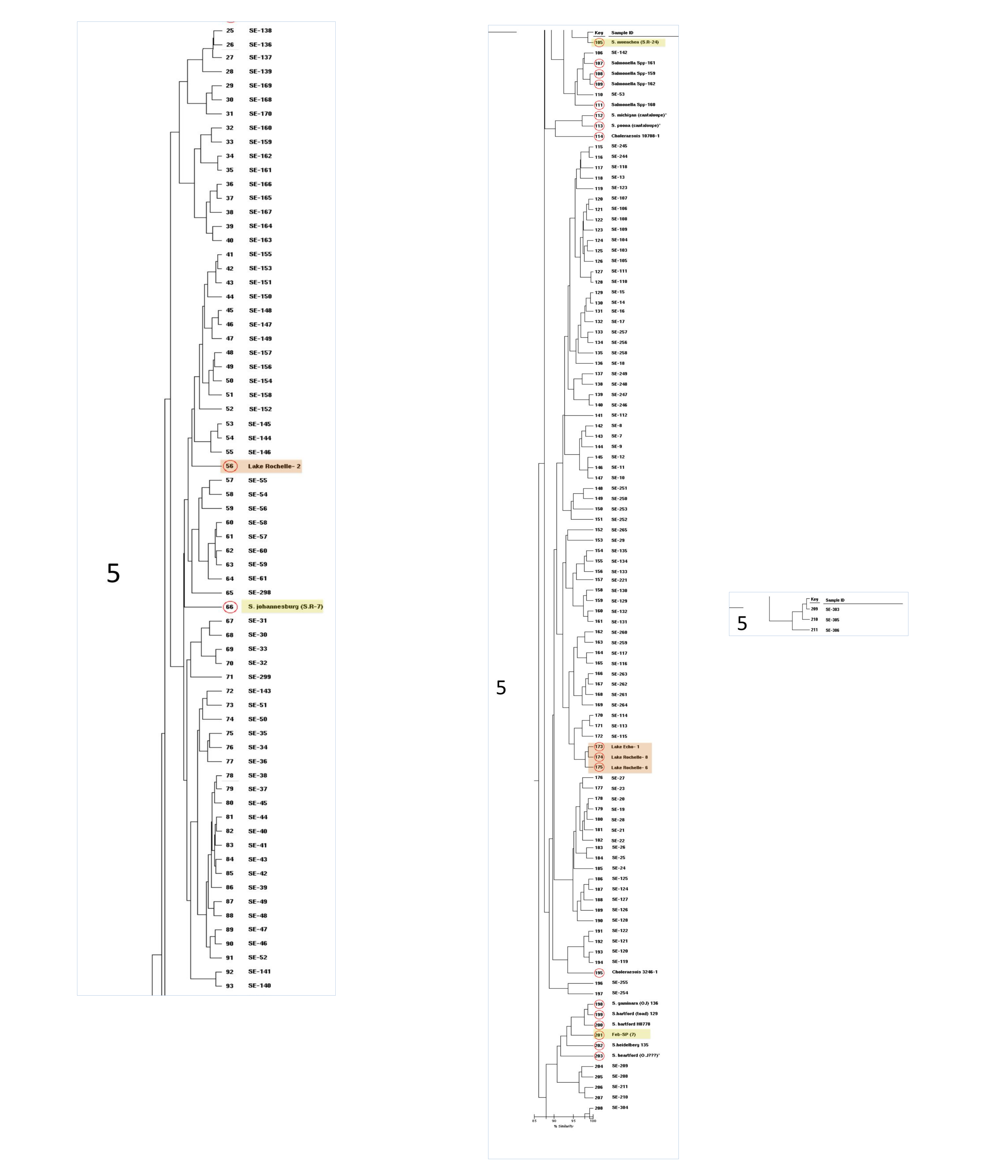

## Slide 4
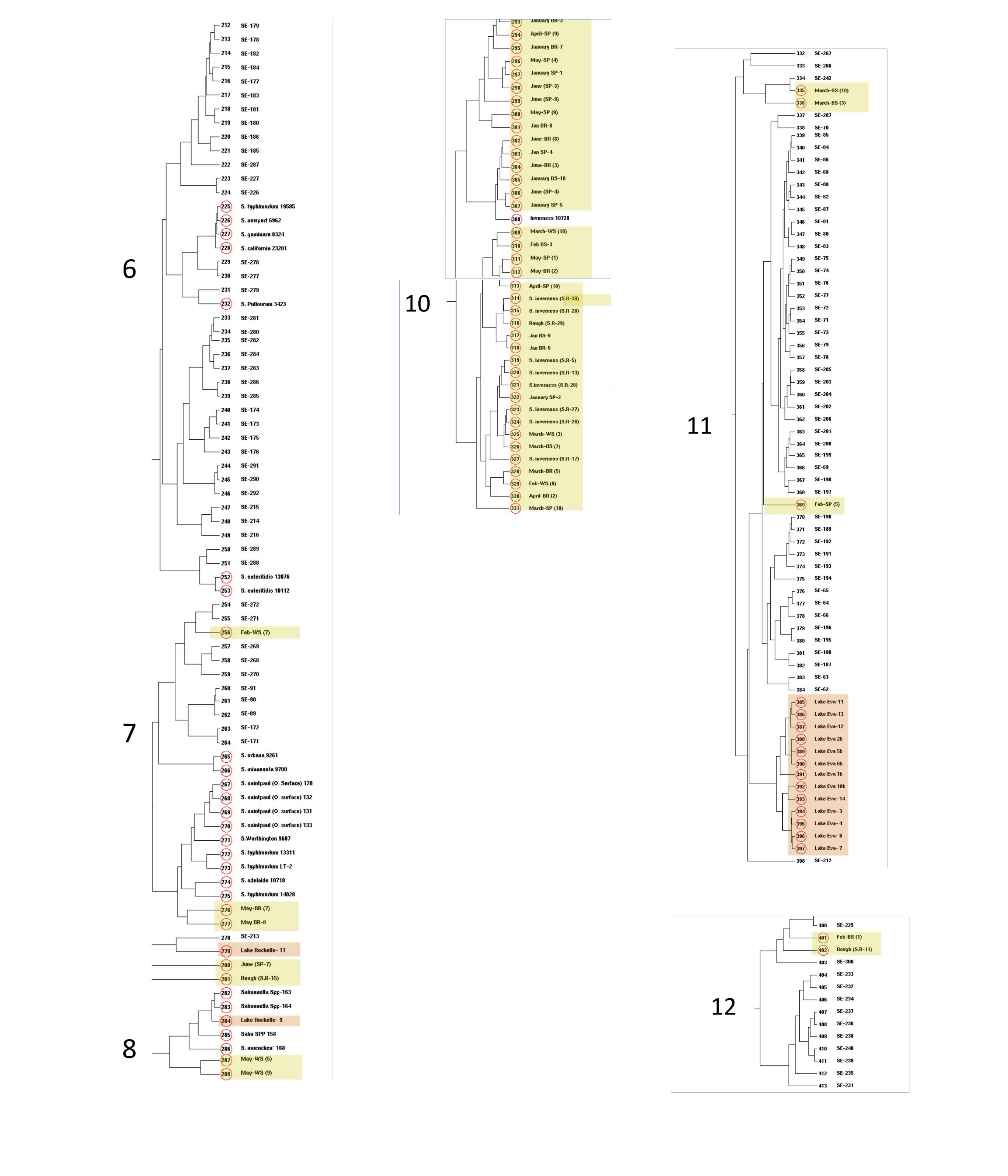

## Slide 5
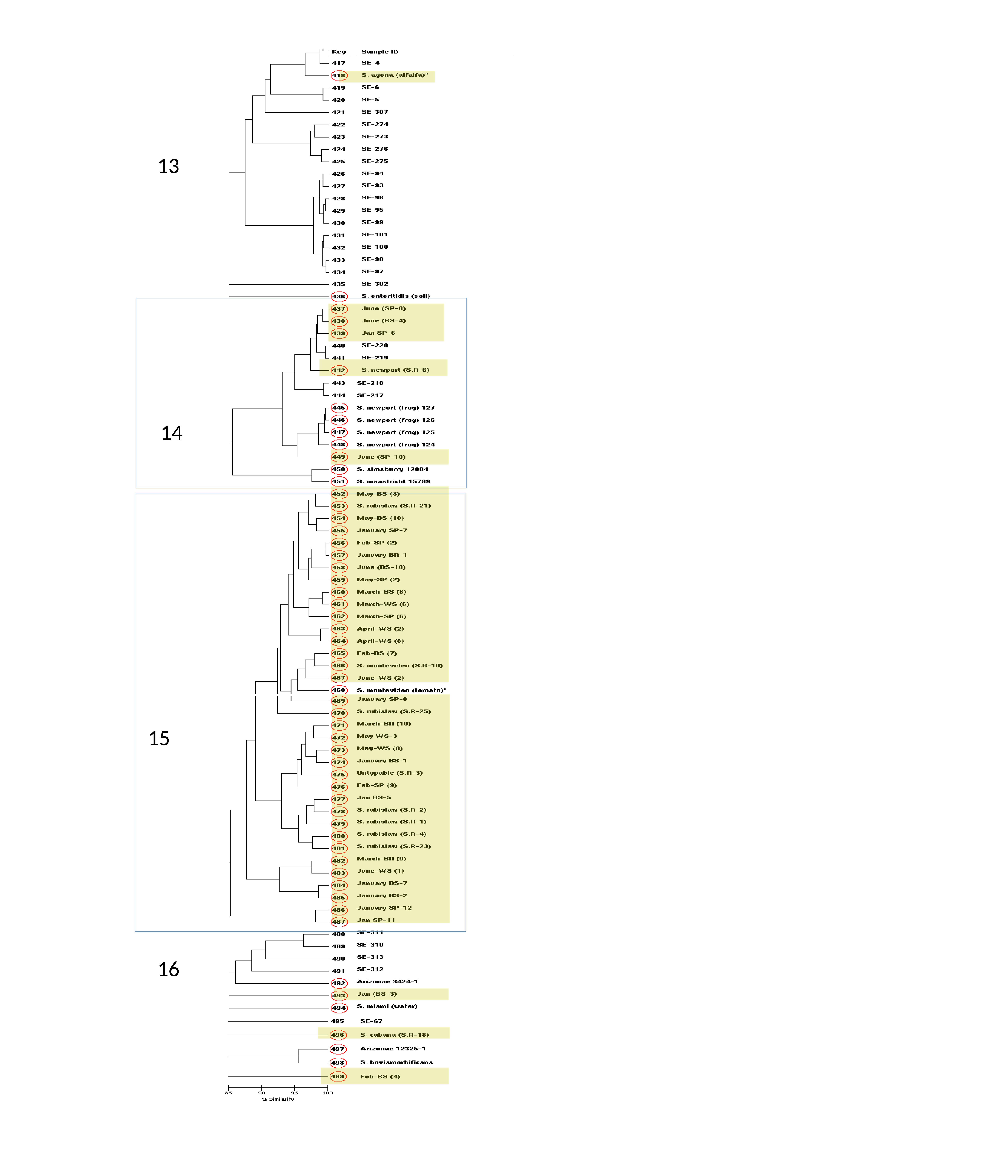

13
14
15
16

Supplement: Supplementary file 1 — Genogroups for Salmonella enterica strains were based on DiversiLab rep-PCR and included Suwannee River isolates (n=110) from this study, which were compared to other environmental (n=47) or clinical (n=28) strains and to an online DiversiLab library (n=314). Analysis of 499 environmental and clinical Salmonella Isolates was assembled into a dendrogram based on rep-PCR fingerprinting profiles at ≥85% DNA similarity level, using DiversiLab software. Genogroups (n=16) were comprised of clusters with >2 strains each. Salmonella isolates from the Suwannee River clustered into 10 genogroups, but the majority of strains were found Genogroups 10 and 15. Legend for Figure 1S should be changed to read “S. enterica genogroups were derived from DiversiLab rep-PCR as described in the Materials and Methods. Strains included Suwannee River isolates from this study (highlighted), which were compared to other environmental or clinical strains. Dendrogram was assembled from rep-PCR fingerprinting profiles at ≥85% DNA similarity level, using DiversiLab software. The strains comprised a total of 16 genogroups with >2 strains each. Gel-like images, serovar identity, isolation source, and identification number for each Salmonella isolate are provided. Abbreviations are used for descriptions of sampling locations on the Suwannee River and include: BS (Big Shoals), WS (White Springs), SP (Spirit of Suwannee), and BR (Boy's Ranch). Additional strains from our collection were from ATCC or provided by Dr. Parish, and strain source is indicated when known. DiversiLab strains are designated by SE number.” [file 461321.f1.zip › 461321.f2.pptx]
